# Bibliometric review on biomarkers for Alzheimer’s disease between 2000 and 2023

**DOI:** 10.1097/MD.0000000000034982

**Published:** 2023-09-08

**Authors:** Xiaojie Yang, Huiling Qu

**Affiliations:** a Department of The Sixth Affiliated Hospital, School of Medicine, South China University of Technology, Foshan, China; b Department of Neurology, General Hospital of Northern Theater Command, Shenyang, China.

**Keywords:** Alzheimer’s disease, bibliometric, biomarkers, CiteSpace, visualization

## Abstract

**Background:**

Alzheimer’s disease (AD) is a common cause of dementia and frailty. Therefore, it is important to develop biomarkers that can diagnose these changes to improve the likelihood of monitoring and treating potential causes. Therefore, this study aimed to examine the relationship between biomarkers and AD, identify journal publications and collaborators, and analyze keywords and research trends using a bibliometric method.

**Methods:**

We systematically searched for papers published in the Web of Science Core Collection database on biomarkers and AD. The search strategy was as follows: (TS) = (Alzheimer’s OR Alzheimer’s OR Alzheimer OR “Alzheimer’s disease” OR “Alzheimer disease”) AND TS = (biomarker OR biomarkers). Only articles and reviews were included as document types, with English as the primary language. The CiteSpace software was used to analyze the retrieved data on countries/regions, institutions, authors, published journals, and keywords. Simultaneously, the co-occurrence of the keywords was constructed.

**Results:**

There were 2625 articles on biomarkers and AD research published by 51 institutions located in 41 countries in 75 journals; the number of articles has shown an increasing trend over the past 20 years. Keywords analysis showed that Alzheimer’s disease, cerebrospinal fluid, mild cognitive impairment, amyloid beta, and tau were also highly influential.

**Conclusion:**

This was the first study to provide an overview of the current status of development, hot spots of study, and future trends in biomarkers for AD. These findings will provide useful information for researchers to explore trends and gaps in the field of biomarkers and AD.

## 1. Introduction

Alzheimer’s disease (AD) is one of the most common causes of dementia and frailty,^[1]^ affecting millions of people worldwide. However, the clinical diagnosis of this condition is often inaccurate. The symptoms of this disease usually begin with mild memory difficulties and develop into cognitive impairment, dysfunction in complex daily activities, and several other aspects of cognition. When AD is clinically diagnosed, several brain regions experience neuronal loss and neuropathic lesions.^[2]^ The key role of suspending potential damage is to promptly administer neuroprotective drugs before mild symptoms transition to AD. To achieve this goal, we need to improve our ability to recognize pre-dementia symptoms.^[3]^ Through early diagnosis, we can better understand the progress of the disease, plan and implement treatment earlier, and monitor reactions to the drugs currently being tested. Traditionally, the diagnosis of dementia is based on medical history, cognitive impairment patterns, and other parameters evaluated through clinical studies, including blood tests and brain structural imaging, to rule out non-degenerative causes of the symptoms. With the prospect of disease improvement, the use of biomarkers to diagnose specific forms of dementia earlier.^[4–6]^ Therefore, it is important to develop biomarkers that can diagnose these changes in order to improve the likelihood of monitoring and treating potential causes. Cerebrospinal fluid (CSF), plasma biomarkers, and amyloid imaging biomarkers can provide information on the neuropathological symptoms of AD.

AD is primarily characterized by amyloid-β (Aβ) protein pathology. Another biomarker of neuronal damage is the tau/phosphorylated tau protein. When 2 biomarkers, Aβ and tau/phosphorylated tau protein, are positively measured, the probability of AD occurrence increases.^[7]^ MRI of medial temporal lobe atrophy, 18FDG-PET of the posterior cingulate and temporoparietal hypometabolism, and amyloid-PET imaging of cortical Aβ deposition are the 3 best-validated neuroimaging biomarkers for AD.^[8]^ Despite insufficient evidence of their clinical practicality, these 3 biomarkers have almost reached analytical and clinical effectiveness. Amyloid-PET is most helpful in excluding AD, whereas 18FDG-PET has value in the differential diagnosis of neurodegenerative diseases, prediction of short-term clinical outcomes, and staging of the range and localization of neurodegenerative processes. Tau-PET is a powerful biomarker for distinguishing tau lesions in AD from other neurodegenerative tau lesions.^[9]^ Tau-PET also helps better understand the role of tau and its relationship with Aβ. Preliminary data indicates that Aβ may accelerate the accumulation of tau and cause tau to diffuse outside the medial temporal lobe.^[10,11]^ Aβ and phosphorylated tau can also be determined using body fluid biomarkers.^[8]^ The established CSF biomarkers are Aβ 1 to 42, Aβ 1 to 40, phosphorylated tau 181, and total tau. Markers reflecting axonal injury and synaptic dysfunction are related to synaptic pathology in the early stages of the disease, and their relationship with functional outcomes and cognitive decline. In some studies, α-synuclein in the CSF has been found in samples of patients with AD or Parkinson’s disease and may be associated with other biochemical biomarkers.^[12]^ Other biomarkers such as neurogranin, SNAP25, synaptotagmins, neuronal calcium sensing protein VLP1, YKL40 (CHI3L1), and soluble TREM2 have emerging^[8]^ in recent years. The level of neurofilament in the plasma is similar to that in the CSF, making the clinical application of this marker feasible. Plasma Aβ and phosphorylated tau 181 and 217 can also serve as diagnostic biomarkers for Alzheimer’s disease and other dementia disorders. Four miRNAs, namely miR-31, miR-93, miR-143, and miR-146a, were observed to be decreased in the serum of patients with AD. Therefore, they have recently been characterized as neoplasm markers of AD pathology and vascular dementia.^[13]^

The early diagnosis of AD may be a cost-effective method to prevent irreversible and uncontrollable consequences. In each case, it is necessary to make a correct diagnosis before the underlying pathology becomes sufficiently severe to manifest clinically. Although many reviews have thoroughly and explicitly addressed biomarkers for AD research, only a few have summarized this topic from the perspective of bibliometric analysis or provided developing trends in this domain.^[14–18]^Some studies performed bibliometric analyses of AD,^[19–24]^ but did not provide future trends of biomarkers for AD. Therefore, we conducted a bibliometric network analysis, which would offer an objective measurement in scientific literature and aggregate the opinions of biomarkers for AD researchers. In this study, we summarized publication trends, highly cited studies, countries/regions, institutions, journals, and authors with the largest contributions and future research directions on the relationship between biomarkers and AD, providing a practical reference for future clinical research work.

## 2. Methods

### 2.1. Search strategy

**Figure 1.**
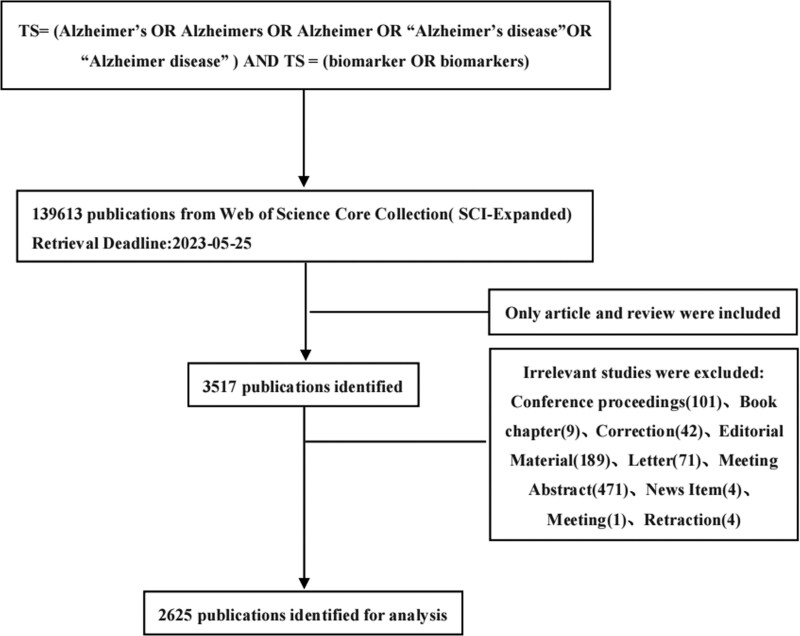
Flow diagram of the publications screening process.

The data used in this study were collected from the science citation index expanded of the Web of Science (WOS) Core Collection database, and were retrieved on May 25, 2023. The search strategy was as follows: (TS) = (Alzheimer’s OR Alzheimer’s OR Alzheimer OR “Alzheimer’s disease” OR “Alzheimer disease”) AND TS = (biomarker OR biomarkers). The search strategy identified papers with these words mentioned in their title. Documents were then filtered by language, including only articles written in English. Only articles and reviews were included as document types. The PRISMA 2020 flow diagram depicts the information flow at different stages of the analysis, as shown in Figure 1. As the data were retrieved from publicly accessible databases, ethical permission was not required.

### 2.2. Analysis methods and tools

The selected documents retrieved by the WOS were imported into the plain text format “txt” files containing all records and references, named “download_XXX” and saved. CiteSpace is one of the most widely used knowledge mapping tools for bibliometric analysis and interactive visualization. Therefore, CiteSpace has proven to be a useful bibliometric method for identifying key points and future trends in the research field. As a supplement, critical reading was used to conduct a more in-depth analysis of key documents to explore the main insights on the topics involved. This study adopted the CiteSpace V.6.1. R6 for scientometric analysis, calculates centrality, and draws co-occurrence maps of countries/regions, institutions, authors, published journals, cited literature, keyword co-occurrence maps, cluster maps, time graphs, and emergent maps. Parameter setting: The overall selection time span was 2000 to May 2023. The slice length was set to 1 year. In CiteSpace, the “*g*-index” is used as a selection criterion, and *k* is a scale factor that is added to the *g*-index calculation and adjusted to include or exclude more nodes. Select Trim, Path Definition, and Trim Network and save the default settings for other options. In the visualization diagram, nodes and font size represent frequency, lines (edges) between nodes represent relationships between nodes, the thickness of lines represents the degree of tightness of relationships, and the colors of nodes and lines represent the distance between years.

## 3. Results

### 3.1. Publication trends

**Figure 2.**
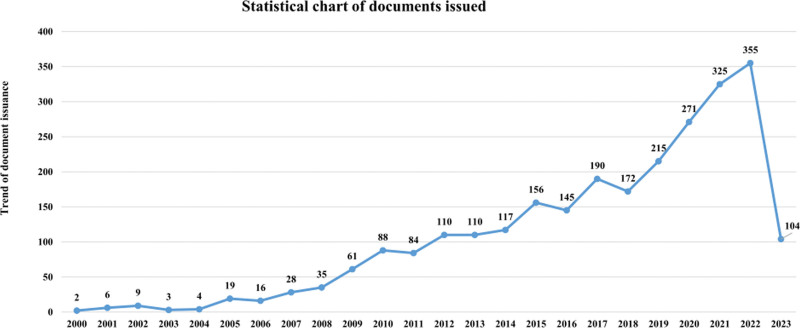
Annual number and growth trend of publications from 2000 to 2023.

A total of 2625 publications met the selection criteria. In recent years, many publications and literature citations have shown the interest of scholars in this field. Consistent with this, despite some minor changes, the overall number of AD and biomarker studies is increasing, as shown in Figure 2. From 2000 to 2005, the number of publications was relatively low at the initial stage, consistently below 10. From 2005 to 2015, the number of publications began to increase yearly, with a relatively steady increase. From then until 2018, there was little fluctuation in the number of publications. From 2019 to 2022, the number of publications gradually increased and reached an explosive trend, peaking in 2022. By less than half a year in 2023, 104 papers had been published, and it is expected that more literature will emerge.

### 3.2. Bibliometric analysis of countries/regions, institutions, journals and author

#### 3.2.1. Analysis of countries.

To further understand the current status of global biomarkers for AD research, we evaluated different countries and regions. The studies were conducted in 41 countries/regions. The earliest and most frequently published country was the United States in 2001, which first published 932 articles, followed by ENGLAND. In 2006, ENGLAND published 395 articles and CHINA published 370 articles since 2011. The other countries with a frequency of over 100 publications were as follows: SWEDEN 352 publications, GERMANY 280 publications, ITALY 231 publications, SPAIN 205 publications, FRANCE 190 publications, NETHERLANDS 171 publications, AUSTRALIA 126 publications, SWITZERLAND 104 publications, BELGIUM 103 publications, CANADA 99 publications, JAPAN and SOUTH KOREA 84 publications, and GREECE 52 publications. Information on the top 15 countries/regions in terms of the frequency of publications is shown in Table 1.

**Table 1:** Top 15 countries/regions in frequency/centrality of publications.

Rank	Frequency	Centrality	Year	Country
1	932	0.45	2001	USA
2	395	0.1	2006	England
3	370	0.1	2011	Peoples R China
4	352	0.12	2005	Sweden
5	280	0.15	2003	Germany
6	231	0.07	2005	Italy
7	205	0.01	2010	Spain
8	190	0.05	2009	France
9	171	0.11	2008	Netherlands
10	126	0.17	2001	Australia
11	104	0.02	2009	Switzerland
12	103	0.01	2007	Belgium
13	99	0.17	2011	Canada
14	84	0.03	2008	Japan
15	84	0	2013	South Korea

#### 3.2.2. Analysis of institutions.

**Figure 3.**
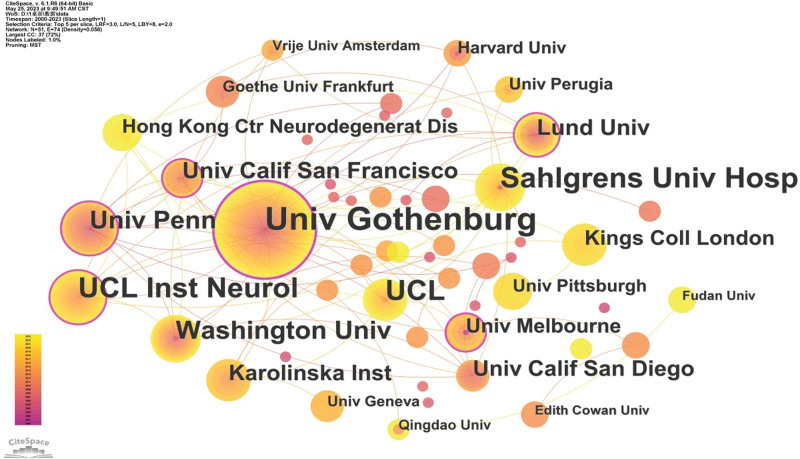
The map of co-institutions. The nodes represent institutes, the number of lines represents the intensity of cooperation between institutions.

A graph analysis of publishing institutions can detect their publication status and cooperation between institutions. A total of 51 nodes (institutions) and 74 connections (cooperative relationships) were obtained from the institutional cooperation diagram, with a density of 0.058. Figure 3 clearly shows the cooperation between institutions. Publishing institutions are mainly concentrated in universities and some are research organizations. The Univ Gothenburg was the most productive institute with the highest frequency(251),followed by Sahlgrens Univ Hosp (136), UCL (125), UCL Inst Neurol (118), Washington Univ (73), Univ Penn (65), Univ California San Francisco (43), Lund Univ(42), Karolinska Inst (39), Univ California San Diego(36), Kings Coll London(29), Univ Melbourne (27), Hong Kong Ctr Neurogenerator Dis (22), Univ Pittsburgh (16), Harvard Univ(15), Univ Perugia (14), Goethe Univ Frankfurt (12), and Univ Geneva (10). The highest centrality is also the Univ Gothenburg (0.56), which suggests that Univ Gothenburg was an authority in this field and had more cooperation with other institutions. From Table 2, it can be seen that in addition to Univ Gothenburg, Sahlgrens Univ Hosp, UCL, UCL Inst Neurol, Washington Univ, Univ Penn, and other nodes also have high centrality, indicating that these institutions have a significant contribution and influence on the academic network.

**Table 2:** Top 15 institutions in frequency/centrality of publications.

Rank	Frequency	Centrality	Year	Institution
1	251	0.56	2006	Univ Gothenburg
2	118	0.18	2013	UCL Inst Neurol
3	42	0.15	2007	Lund Univ
4	65	0.13	2006	Univ Penn
5	43	0.12	2010	Univ Calif San Francisco
6	27	0.11	2001	Univ Melbourne
7	73	0.08	2005	Washington Univ
8	39	0.08	2014	Karolinska Inst
9	136	0.07	2008	Sahlgrens Univ Hosp
10	2	0.06	2006	Malmo Univ Hosp
11	6	0.06	2013	Austin Hlth
12	125	0.06	2014	UCL
13	4	0.06	2014	Univ London Imperial Coll Sci Technol & Med
14	29	0.06	2020	Kings Coll London
15	15	0.01	2006	Harvard Univ

#### 3.2.3. Analysis of journals.

Academic journals are the media for exchanging and disseminating knowledge in various disciplines. According to the data analysis, 75 journals were published as biomarkers for AD. Table 3 lists the top 10 most highly cited journals in this area. The highest-cited journal is *Neurology*, with 2015 citations and a centrality of 0.11, followed by *Alzheimers Dement* (1887), with a centrality of 0.13. The remaining journals with more than 1000 citations were listed in the following order: *Jalzheimers Dis* (1823), *Neurobiol Aging* (1755), *Lancet Neurol* (1610), *Ann Neurol* (1437), *Arch Neurol-Chicago* (1399), *Brain* (1221), *Plos One* (1055), and *P Natl Acad Sci USA* (1040). Most highly cited journals have high centrality in this field, indicating that these journals are the core of this research field.

**Table 3:** Top 10 journals in frequency/centrality of publications.

Rank	Citations	Centrality	Year	Journal
1	2015	0.11	2001	*Neurology*
2	1887	0.13	2009	*Alzheimers Dement*
3	1823	0.18	2002	*J Alzheimers Dis*
4	1755	0.42	2001	*Neurobiol Aging*
5	1610	0.23	2005	*Lancet Neurol*
6	1437	0.15	2000	*Ann Neurol*
7	1399	0.1	2000	*Arch Neurol-Chicago*
8	1221	0.03	2007	*Brain*
9	1055	0	2013	*Plos One*
10	1040	0.14	2000	*P Natl Acad Sci USA*

#### 3.2.4. Analysis of authors.

**Figure 4.**
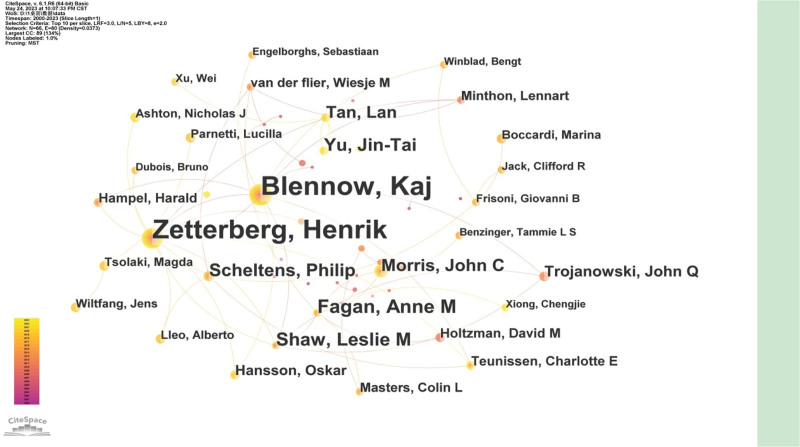
The map of coauthors. The nodes in the map represent coauthors, and lines between the nodes represent co-citation relationships.

There are 66 authors who have contributed to biomarkers for AD research in the WOS Core Collection. The researchers and their collaborations regarding biomarkers for AD are shown in Figure 4. We can see that the collaboration was very close with many clusters. The top 3 authors who published a large number of publications were Blennow, Kaj (214), Zetterberg, Henrik (213), Fagan, and Anne M (43), and the other authors with a frequency of publication over 20 are ranked as follows: Shaw, Leslie M (37), Yu, Jin Tai (33), Scheltens and Philip (31), Morris, John C (27), and Tan and Lan (26). The large-scale author collaboration teams led by Zetterberg and Henrik as well as other research collaborations between authors in this field are presented in the form of connections between nodes in Figure 4. Some authors who only have nodes but no connections have independently researched and published articles, such as Hansson, Oskar, Holtzman, David M, Hampel, and Harald.

### 3.3. Research topic analysis

#### 3.3.1. Analysis of co-occurrence keywords.

The frequency of occurrence of the keyword “Alzheimer’s disease” is the highest, with 1397 times, followed by “mild cognitive impairment,” with a frequency of 918 times. In addition, the frequency of “cerebrospinal fluid” is 766 times, “dementia” is 748 times, “national institute” is 373 times. Information on the top 15 keywords, in descending order, is summarized in Table 4.

**Table 4:** Top 15 keywords in cited times.

Rank	Citations	Centrality	Year	Keyword
1	1397	0.56	2002	Alzheimer’s disease
2	918	0.67	2005	Mild cognitive impairment
3	766	0.43	2002	Cerebrospinal fluid
4	748	0.26	2005	Dementia
5	373	0.09	2012	National institute
6	308	0.05	2006	Diagnosis
7	241	0.06	2012	Association workgroup
8	226	0.08	2010	Amyloid beta
9	211	0.11	2008	Tau
10	188	0	2009	Association
11	168	0.04	2007	Brain
12	153	0.05	2009	CSF biomarker
13	116	0	2012	Recommendation
14	114	0.01	2005	Protein
15	111	0	2014	Diagnostic guideline

**Figure 5.**
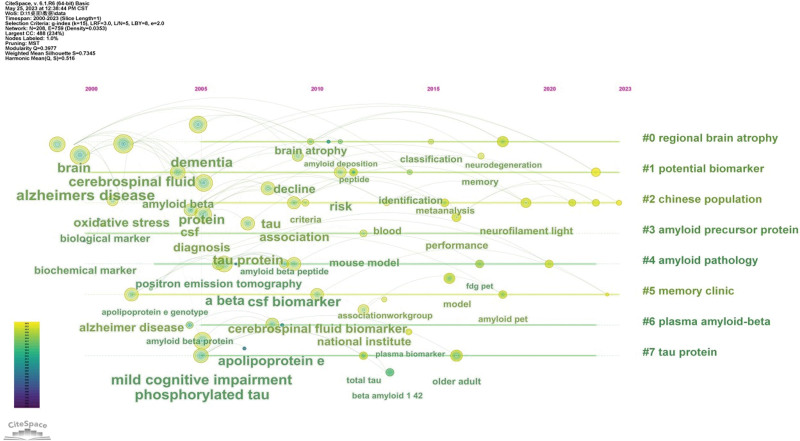
The time diagram of keywords.

Keyword clustering can indicate different research focuses in the field, and we extracted these 8 clusters for the timeline analysis (Fig. 5). The cluster numbers are # 0 to # 7. The larger the number of clusters, the fewer keywords contained in the cluster; the smaller the number, the more keywords included in the cluster. These keywords were spread out within their respective clusters based on their year of occurrence, displaying the development of keywords within each cluster in Figure 5. The clustering label words were: #0, regional brain atrophy; #1, potential biomarker; #2, Chinese population; #3, amyloid precursor protein; #4, amyloid pathology; #5, memory clinic; #6, plasma amyloid beta; and #7, tau protein. Between 2000 and 2015, there were a large number of hot keywords in related fields that were in the explosive period of research. Subsequently, the heat of the research stabilized. From late 2015 to 2023, the number of keywords appearing decreased and research in this field experienced a decline.

#### 3.3.2. Future research direction analysis.

**Figure 6.**
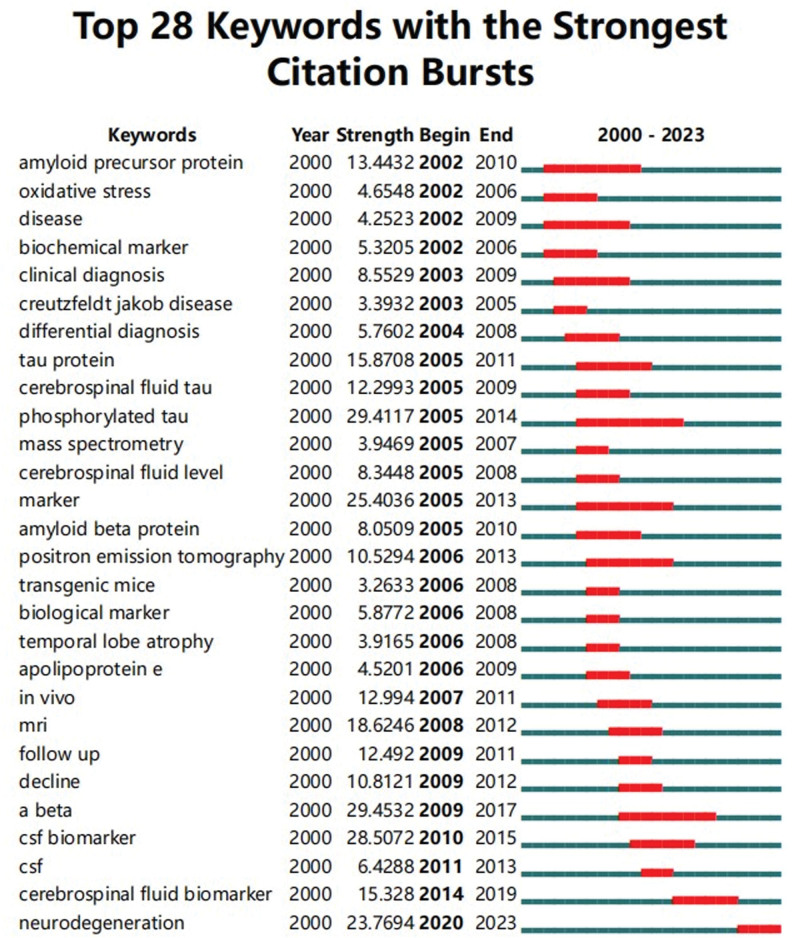
The emergent map of keywords. The red horizontal stripes represent the years with the most frequent keywords. The green horizontal stripes represent the years with the most infrequent keywords.

The time span of the theme evolution was from 2000 to 2023 (Fig. 6). From the time shown in Figure 6, it can be seen that there were no prominent keywords between 2000 and 2002. By 2000, the term “amyloid precursor protein” had begun to emerge and continued until 2010. In the same year, the hot words “oxidative stress” and “biological marker” emerged, and their popularity continued until 2006. The word “disease” continued until 2009 and lasted for a longer period of time. In addition, “a beta” emerged in 2009 and continued until 2017, with the highest intensity of the keyword emergence reaching 29.4532. The “photosphylated tau” in 2005 took second place of intensity, reaching 29.4117, and continued until 2014. In 2010, the intensity of the word “CSF biomarker” in 2015 reached 28.5072. In 2005, the emergence of the word “marker” continued until 2013, and the emergence intensity reached 25.4036. The emergence of the word “neuron generation” lasted until 2023, with an intensity of 23.7694. Words with high emergence intensity and long duration are still a research hotspot in this field.

## 4. Discussion

Using bibliometric and visual analysis methods, this study discussed research trends and hotspots in the field of AD and biomarkers from 2000 to 2023. According to this study, AD and biomarker research have shown an increasing trend over the past 20 years, with 2022 being the year with the most published literature. The number of published papers in 2022 is 4.03 times that in 2010 and 177.5 times that in 2000.

Among the top 15 most productive countries that published most of the publications, the United States not only had the largest output in publications, the first in the centrality ranking, but also the earliest country to publish articles. The publication frequency of the United States is more than twice that of the second-ranked England and the third-ranked China. The United States has laid the foundation for the development of this field and is an authority in this field. However, University Gothenburg, which has the highest frequency and centrality from Sweden, has conducted more in-depth research and made more significant contributions to this field than other institutions. It is worth noting that the second highest frequency of institutes is also from Sweden. Although Sweden ranks 4th in terms of publication frequency, it ranks first and second among the research institutions. The core author, Blennow, Kaj, with the highest number of publications in this field, also comes from this institute, indicating that Sweden led biomarkers and AD research. However, China ranks third in terms of publication frequency, and lacks influential institutions. China should focus on developing influential research institutions in the future. The majority of publications come from developed countries, and it is very important to improve the prevention, diagnosis, treatment, and care of dementia patients in low- and middle-income countries and regions by drawing on the experience of intervention measures and services from developed countries on the basis of improving awareness and diagnostic levels.

By analyzing journals related to biomarkers and AD, we determined that researchers are concentrated in the fields of neuroscience and geriatrics, especially in AD specialist journals. The highest-cited journal in this field is Neurology, which is the most widely read and highly cited peer-reviewed neurology journal with a high impact factor. Neurology is the official journal of the American Academy of Neurology, indicating the leading role of the United States in this field. Alzheimer’s Dement, the second most cited journal in this field, is also a top-level journal in the field of neuroscience with an impact factor of 16.655 from Q1 journals. This means that the publication quality of these journals is far higher than the average level, and that this field is also a current research hotspot, receiving attention from top journals. In addition, it is worth noting that there are relatively more studies in this field. We believe that these high-quality and extensive research results with innovative and groundbreaking discoveries confirm the importance of biomarkers for AD, and actively promote research and development in this field.

In a certain research field, keyword co-occurrence can be used to classify high-frequency keywords and study the strength of relationships between keywords by examining the co-occurrence of keywords in a large number of publications. There is no doubt that the keyword “Alzheimer’s disease” appears most frequently in this field. Keywords analysis showed that “cerebrospinal fluid,” “mild cognitive impairment,” “amyloid beta,” and “tau” were highly influential. Biomarkers in the CSF are valuable for determining disease diagnosis and predicting outcomes. They also play important roles in the development of new drug therapies.^[25]^ The existing biomarkers used for diagnosing AD include; 42 amino acid forms of amyloid β in the CSF; Total and phosphorylated tau proteins in CSF.^[26]^ Aβ combined with *p* tau and *t* tau in the CSF has been established as an acceptable biomarker for the diagnosis of AD. Aβ in the CSF helps distinguish between AD and other neurodegenerative diseases.^[27,28]^ The *t* tau and phosphorylated tau of CSF (located at threonine 181), denoted as *t* tau or *p* tau, have been comprehensively studied as general biomarkers of neuronal damage in neurodegeneration.^[4]^ In 2011, the National Institute on Aging and Alzheimer’s Association released research standards that included a combination of clinical features and biomarkers such as *t* tau and *p* tau for the diagnosis of mild cognitive impairment (MCI) caused by AD.^[29]^ MCI is used to describe the early stages of AD, and identifying and treating it before further decline is an important clinical task. MCI involves mild but significant degradation of cognitive skills, including thinking skills and memory, and is a risk factor for the development of AD.^[30]^ CSF *t* tau has been proven to be a reasonable prognostic indicator for progression from cognitive impairment to MCI, followed by AD.^[31]^ Patients with MCI and lower levels of CSF Aβ showed faster progression to AD. Early recognition of MCI by identifying Aβ and tau in CSF has become a research hotspot. According to the cluster analysis results, studies related to “regional brain atrophy” have attracted wide attention. These findings indicate that, in addition to the cerebrospinal fluid proteins discussed, regional magnetic resonance imaging measurement of brain volume atrophy is also an important biomarker related to AD. The “Chinese population” has also become a label keyword, which indicates that Chinese scholars are increasingly paying attention to research on the identification of patients with AD in China with the advent of an aging society.

As the keywords with the strongest citation bursts after 2000, we found that “CSF biomarker,” “CSF,” “cerebrospinal fluid biomarker,” and “neurodegeneration” have been the research frontier since 2010. In the past decade, significant progress has been made in the study of biomarkers in the CSF of patients with AD. With the exception of classic and core biomarkers such as Aβ and tau for diagnosing AD, several new biomarkers for AD pathogenesis have been identified in the past decade.^[32]^ These biomarkers can provide higher diagnostic and prognostic accuracy in research cohorts and better understand the changes in neuropathology during development. Studying these new CSF biomarkers and their relationship with the classic AD triad may indicate further developments in clinical applications. To promote the differential diagnosis and prognosis of AD compared with other diseases, these CSF biomarkers provide satisfactory differentiation prospects for AD.

## 5. Limitations

When interpreting the results of this study, several scientific factors should be considered. First, we only searched for literature from the WOS core collection database, as different databases have different attributes such as citation count and export format. Second, English papers accounted for the papers included in our research, as the WOS database mainly indexed English papers, which may have led us to overlook relevant research published in other languages. In addition, new publications may subsequently be added to the May 2023 queue; however, the database was not updated during retrieval. However, this section of the literature is limited and does not cause significant errors in the analysis.

## 6. Conclusion

To the best of our knowledge, this is the first study to provide an overview of the current status of development, hot spots of study, and future trends in biomarkers for AD. Overall, the analysis using the CiteSpace software showed co-occurrence maps of countries/regions, institutions, authors, published journals, cited literature, keyword co-occurrence maps, cluster maps, time graphs, and emergent maps from 2000 to 2023. The number of publications on biomarkers and AD has been increasing rapidly, especially in the past 3 years. Most of these publications are associated with neuroscience and geriatrics, but they also involve biomarkers and cognition. From this perspective, enhanced interagency and interdisciplinary cooperation is essential for the progress and development of this scientific field. The United States, dominate in terms of publication and research collaboration on biomarkers for AD. The other countries need to actively seek international cooperation to enhance their global influence for the further development of this field. “Cerebrospinal fluid,” “mild cognitive impairment,” “amyloid beta,” and “tau” are considered the current research hotspots and frontiers in this field. We believe that these findings will provide useful information for researchers to explore trends and gaps in the field of biomarkers and AD.

## Author contributions

**Writing – review & editing:** Huiling Qu.

**Writing – original draft:** Xiaojie Yang.
